# Bioinformatics Identification of the Expression and Clinical Significance of E2F Family in Endometrial Cancer

**DOI:** 10.3389/fgene.2020.557188

**Published:** 2020-11-04

**Authors:** YunZheng Zhang, Zihao Wang, Jian Ma, JiaNing Huo, YiBing Li, YuShan Wang, Hao Chen, LuHe Shan, Xiaoxin Ma

**Affiliations:** Department of Obstetrics and Gynecology, Shengjing Hospital of China Medical University, Shenyang, China

**Keywords:** endometrial cancer, E2Fs, biomarker, bioinformatics analysis, immune infiltration

## Abstract

**Background:**

Besides being one of the most prevalent cancers among women, incidence and mortality rates of endometrial cancer (EC) are still increasing. The E2F family of transcriptional factors is involved in cell differentiation, apoptosis, and inhibition of DNA damage response, thus affecting growth and invasion of tumor cells.

**Methods:**

We used multiple bioinformatics tools to explore the role of E2F family in endometrial cancer.

**Results:**

The expression of E2F1/2/3/7/8 was significantly upregulated in endometrial cancer tissues, converse to E2F4, which was downregulated. Methylation downregulates all E2Fs except for E2F2. Accordingly, E2F1/2/3/5/7/8 are potential diagnostic biomarkers for EC. In particular, EC patients displaying upregulated E2F1, and E2F3 expression had a worse overall survival and relapse-free survival. E2F8, E2F7, and E2F1 were the top three, most-frequently altered genes in endometrial cancer. E2F family activates apoptosis pathways, regulates cell cycle, and impairs DNA damage response pathways. Drug-sensitivity analysis demonstrated that the level of E2F2/3/8 negatively correlated with drug resistance. Meanwhile, immune infiltrations analysis revealed that E2F family is associated with recruitment of several immune cells. Enrichment analysis on its part revealed that the E2F family is mainly associated with cell cycle, sequence-specific DNA binding, nuclear transcription factor complex, PI3K-Akt signaling, and p53 signaling pathway. We also identified multiple E2Fs-associated miRNA and kinase targets in endometrial cancer.

**Conclusion:**

Our study revealed the unique expression signature and clinical significance of E2F family in EC, demonstrating the potential clinical utility of these transcription factors (TF) in endometrial cancer.

## Introduction

Endometrial cancer (EC) is one of the most prevalent cancers among women. It is estimated that in US alone, 65,620 women will be diagnosed with EC, of which 12,590 of them will die of this disease in 2020 (Siegel et al., 2020). These bleak statistics notwithstanding, the incidence and mortality rate due to EC are all increasing. In fact, for the past few years, the mortality rate has increased more rapidly than the incidence rate of EC (Morice et al., 2016; Rose et al., 2017; Arend et al., 2018). The 5-year overall survival (OS) rate for early stage EC is 91%, but drops to 74% when the disease exhibits metastasis (Morice et al., 2016). The therapeutic options for EC, particularly for advance stage types, are limited. Confounding challenges associated with advanced stage and metastatic EC underlines the unmet need for accurate diagnostic, prognostic, and therapeutic biomarkers for EC.

The E2F family are a group of transcription factors (TFs) that regulate gene expression by modulating the initiation and transition of G1/S and G2/M cell cycles (Rösner and Sørensen, 2019). In mammals, eight members of the E2F family (E2F1–E2F8) have been identified. Increasing evidences show that members of the E2F family participate in cell differentiation, apoptosis, and DNA damage response, thus impacting growth and invasion of tumor cells (Trimarchi and Lees, 2002). Members of the E2F family have also been suggested to be diagnostic and prognostic biomarkers for certain cancers, including breast and gastric cancer (Liu Z. L. et al., 2018; Manicum et al., 2018).

However, the expression profile and their specific functions of E2Fs in EC remain unclear. In this study, we systematically assessed the expression, diagnostic, and prognostic values of E2Fs. We further analyzed their regulatory networks in EC. Findings of this research will provide additional but much-needed evidences on the role of E2Fs in tumorigenesis, progress diagnostic and prognostic potential of these TF in EC.

The development of the second-generation gene-sequencing technology and large consortium projects such as The Cancer Genome Atlas (TCGA) database have created new opportunities for data mining and deeper understanding of gene functions, including tumors associated genes. In this study, we used various TCGA visualization computational tools including UALCAN, GEPIA, Kaplan Meier-plotter, GSCALite, cBioPortal, TIMER, LinkedOmics, GeneMANIA, DAVID V 6.8, and Metascape to assess the role of E2Fs in EC. The flow chart of the current is shown in Supplementary Figure 1.

## Materials and Methods

### UALCAN

We first explored the expression of E2Fs in EC using UALCAN (http://ualcan.path.uab.edu/index.html, a bioinformatics tool developed by Chandrashekar et al. (Chandrashekar et al., 2017). UALCAN is a bioinformatics tool for analyzing and visualizing TCGA. Thereafter, we used the same tool to analyze the expression of E2F family and its correlation with multiple clinical pathological parameters, including tumor grade, cancer stage, race among other factors, in EC. The analyses were performed on data extracted from the TCGA_UCEC (Uterine corpus endometrial carcinoma, *n* = 546) dataset *P*-value < 0.05 was considered statically significant.

### Specimens and Patients of Quantitative Real-Time Polymerase Chain Reaction (qRT-PCR)

A total of 27 EC tissues and 20 normal endometrial tissues were obtained from patients who underwent a hysterectomy or endometrial curettage for endometrial-irrelevant diseases in Shengjing Hospital of China Medical University, China, from 2017 to 2019. All patients provided informed consent, and this study was approved by the Ethics Committee of Shengjing Hospital of China Medical University. Histological diagnosis and tumor grade were assessed by three experienced pathologists in accordance with the International Federation of Gynecology and Obstetrics (FIGO 2009). None of the patients received any local or systemic treatment preoperatively.

Total RNA of EC tissues and normal endometrial tissues were extracted with TRIzol reagent (Vazyme, Nanjing, China). The synthesis of cDNAs corresponding to the mRNAs of interest depended on PrimeScript RT-polymerase (Vazyme). SYBR-Green Premix (Vazyme) with specific PCR primers (Sangon Biotech Co., Ltd., Shanghai, China). Glyceraldehyde-3-phosphate dehydrogenase was used as an internal control. The 2-ΔΔCt method was used to calculate fold-changes. Primer sequences are shown in Supplementary Table 1. The difference of the expression of E2Fs in tumor tissues and normal tissues were evaluated with Student’s *t*-test in GraphPad Prism7 software (GraphPad, Inc., La Jolla, CA, USA).

### GEPIA

GEPIA (http://gepia.cancer-pku.cn/about.html) is a web-based bioinformatics tool developed by Tang, Z. et al. (Tang et al., 2017), utilized for fast and customized analysis of gene sets. We performed differential expression analysis of RNA sequences extracted from TCGA and the GTEx datasets. GEPIA is a bioinformatics tool for analyzing and visualizing TCGA. GEPIA facilitated the analysis of relative expression of E2Fs between UCEC and normal tissues. The correlation between each member of E2F family and UCEC was also performed using the same tool, based on TCGA_UCEC (*n* = 546) dataset in GEPIA.

### Kaplan Meier-Plotter

Kaplan Meier-plotter (KM-plotter, http://www.kmplot.com/) is a also an online bioinformatic tool developed for comprehensive prognosis analyses (Hou et al., 2017). KM-plotter is a bioinformatics tool for analyzing and visualizing TCGA. It was utilized to explore the prognostic value of E2Fs in patients with UCEC. Here, the patients were stratified into low or high expression group, with the median value of E2F family set as the cutoff score. The same tool was utilized in generate the OS and relapse-free survival (RFS) curves for E2Fs in UCEC, based on the previously described data. A *p*-value < 0.05 was considered statically significant.

### GSCALite

GSCALite (http://bioinfo.life.hust.edu.cn/web/GSCALite/) is another bioinformatics tool, developed by An-Yuan Guo et al. (Liu C. J. et al., 2018). GSCAlite is a bioinformatics tool for analyzing and visualizing TCGA. It was utilized in assessing the Methylation module of E2Fs under UCEC, and the corresponding correlation of gene expression and patients’ survival. Differences in the methylation patterns between UCEC and normal tissues were assessed using Student *t*-test. The *p*-value was adjusted by false discovery rate (FDR), with TFDR < 0.05 considered to be statistically significant. The frequency and variant types of E2Fs in UCEC were displayed graphically using single nucleotide variation (SNV). Meanwhile, a summary of SNV and Oncoplot waterfall plot was generated using maftools (Mayakonda and Koeffler, 2016). The correlation between members E2F family and pathway activity were assessed using Pathway Activity module. The pathway scores were awarded as previous described (Ye et al., 2018). The correlation between members of E2F family and drug sensitivity was analyzed using the drug-sensitivity module. The linear correlation between the expression of E2Fs and the 265 small molecules from Genomics of Drug Sensitivity in Cancer (GDSC) was analyzed using the Pearson correlation coefficient.

### cBioportal

cBioportal (https://www.cbioportal.org/) is another bioinformatics tool developed for comprehensive genomics analysis (Zeng et al., 2019). cBioportal is a bioinformatics tool for analyzing and visualizing TCGA. In particular, the platform was used to analyze 546 UCEC in TCGA_dataset patients. The mRNA expression z scores (RNA Seq V2 RSEM) were obtained using a z score threshold of ±2.0. Protein expression z scores (RPPA) were obtained using a z score threshold of ±2.0. “Survival” module explored the effects of genetic alteration E2F members on the prognosis of patients. Meanwhile, the “Network” module explored the correlation between E2Fs and genes adjacent to UCEC.

### TIMER

TIMER (http://cistrome.org/TIMER/) is a bioinformatics tool developed for analysis of immune infiltrations (Li et al., 2016). TIMER is a bioinformatics tool for analyzing and visualizing TCGA. It was used to explore the correlation between members of E2F family and infiltration of immune cells in UCEC. Significance difference in somatic copy number in relation to alterations in E2F genes was analyzed using a two-sided Wilcoxon rank-sum test. The analysis was performed based on the TCGA-UCEC dataset (*n* = 546). *P*-value < 0.05 was considered statically significant.

### Enrichment Analysis

Enrichment analysis of E2Fs in UCEC was performed using GeneMANIA, DAVID V 6.8 and Metascape (Zeng et al., 2019). We first established significant genes adjacent to E2Fs using GeneMANIA correlation genes analysis. Thereafter, E2F gene family and neighboring associated genes were then analyzed using DAVID 6.8 and Metascape for the GO and KEGG pathways. The results were visualized with R project using a “ggplot2” package and a *p* < 0.05. Biological processes (BPs), cellular components (CCs), and molecular function (MF) were all assessed in the GO enrichment analysis. In Metascape, the analysis was performed with min overlap of 3, *P*-value of 0.01, and min enrichment of 1.5.

### LinkedOmics

LinkedOmics (http://www.linkedomics.org/) is a bioinformatics tool that provides comprehensive multiomics data analysis across 32 TCGA cancer types (Zeng et al., 2019). LinkedOmics is a bioinformatics tool for analyzing and visualizing TCGA. The “LinkInterpreter” module of LinkedOmics was used to derive biological insights into kinase and miRNA target for E2F family in UCEC. Gene Set Enrichment Analysis (GSEA) was performed with a minimum number of genes (size) of 3 and a simulation of 500, within the TCGA UCEC dataset (*n* = 546). Results were analyzed statistically using the Spearman correlation test.

## Results

### Defining E2F Family in UCEC

**FIGURE 1:**
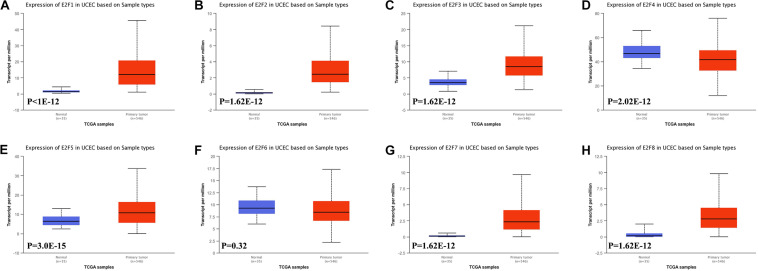
The level of E2Fs in normal and UCEC tissues. Box plots showed the expression of E2F1 **(A)**, E2F2 **(B)**, E2F3 **(C)**, E2F4 **(D)**, E2F5 **(E)**, E2F6 **(F)**, E2F7 **(G)**, and E2F8 **(H)** in UCEC tissue and normal tissues.

**FIGURE 2:**
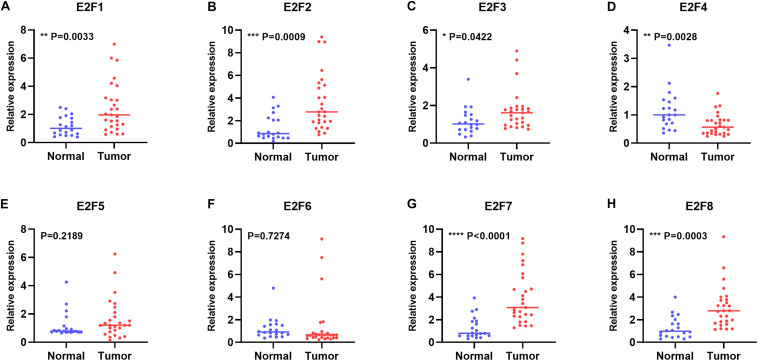
The relative level of E2Fs in normal and UCEC tissues. Scatter plots showed the relative level of E2F1 **(A)**, E2F2 **(B)**, E2F3 **(C)**, E2F4 **(D)**, E2F5 **(E)**, E2F6 **(F)**, E2F7 **(G)**, and E2F8 **(H)** in UCEC tissue and normal tissues.

In this study, we first explored expression of E2Fs in UCEC using UALCAN. Our analysis revealed significant over-expression of E2F1 (Figure 1A, *P* < 1E-12), E2F2 (Figure 1B, *P* = 1.62E-12), E2F3 (Figure 1C, *P* = 1.62E-12), E2F5 (Figure 1E, *P* = 3.0E-15), E2F7 (Figure 1G, *P* = 1.62E-12), and E2F8 (Figure 1H, *P* = 1.62E-12) in UCEC tissues, converse to E2F4 (Figure 1D, *P* = 2.02E-12), which was downregulated in the same tissues. We then verified the expression E2Fs in UCEC using qRT-PCR. As a result, the expression of E2F1 (Figure 2A, *P* = 0.0033), E2F2 (Figure 2B, *P* = 0.0009), E2F3 (Figure 2C, *P* = 0.0422), E2F7 (Figure 2G, *P* < 0.0001), and E2F8 (Figure 2H, *P* = 0.0003) were upregulated in UCEC tissues while E2F4 (Figure 2D, *P* = 0.0028). However, there is no difference between UCEC tissues and normal tissues in the expression of E2F5 (Figure 2E, *P* = 0.2189) and E2F2 (Figure 2F, *P* = 0.7274). Thus, we suggested that the expression of E2F1/2/3/7/8 was significantly upregulated in EC tissues, converse to E2F4, which was downregulated.

**FIGURE 3:**
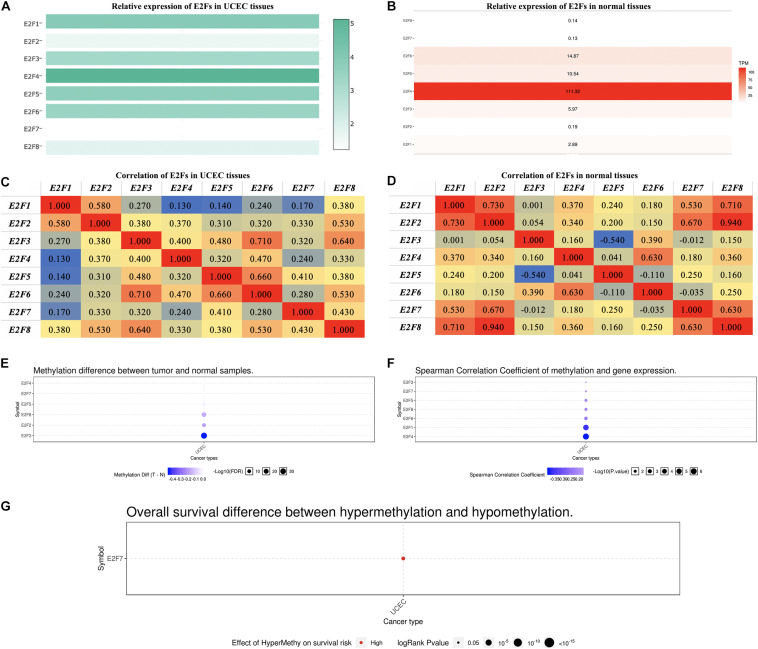
The expression of E2Fs in normal and UCEC. **(A)** The relative expression of E2Fs in UCEC tissues and **(B)** normal tissues. **(C)** Correlation between each E2F member and UCEC and **(D)** normal tissues. **(E)** Differences in the methylation of E2Fs in UCEC and normal tissues. **(F)** Correlation between methylation of E2Fs and their corresponding expression in UCEC. **(G)** The role of methylated E2F7 in the survival of UCEC patients.

In exploring the relative level of E2F in UCEC and normal tissues, overexpression of E2F4 ranked first, with E2F7 being the lowest expressed member in UCEC tissues (Figure 3A). In normal tissues, E2F4 was relatively the highest expressed gene, with E2F8 being the lowest (Figure 3B). Regardless of the tissues, there was always a low to high correlation between each member of E2F family and normal or UCEC tissues (Figure 3C,D).

Methylation analysis revealed that E2F2, E2F3, and E2F8 were not methylated in UCEC tissues (Figure 3E). Interestingly, methylation downregulates all the members of E2F family except for E2F2 (Figure 3F). We also observed that E2F7 hypermethylation is a risk factor for developing UCEC (Figure 3G).

### The Association Between E2Fs and UCEC Clinical Pathological Features

**FIGURE 4:**
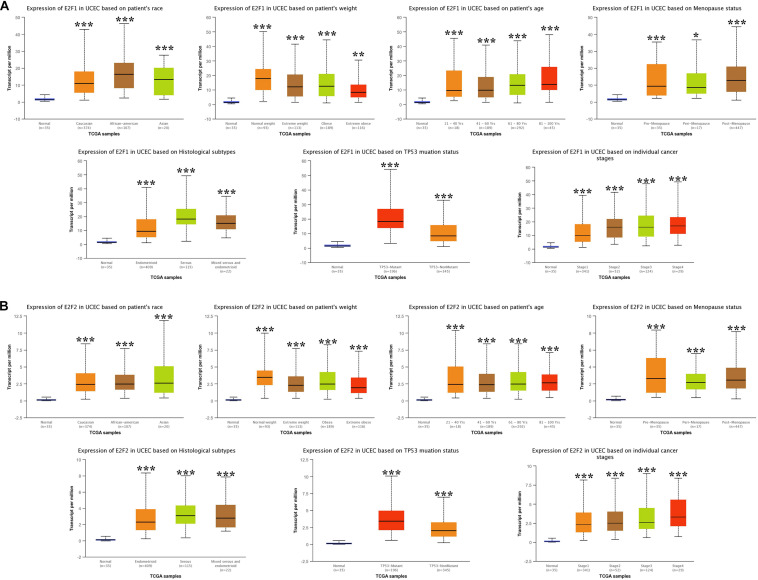
The level of E2F1 and E2F2 in UCEC. **(A)** E2F1 expression in multiple subgroups of patients with UCEC (race, weight, age, menopause status, histological subtypes, TP53 mutation status, and cancer stage). **(B)** Expression of E2F2 in multiple subgroups of patients with UCEC. Stratified based on race, weight, age, menopause status, histological subtypes, TP53 mutation status and cancer stage. *P < 0.05; **P < 0.01; ***P < 0.001).

**FIGURE 5:**
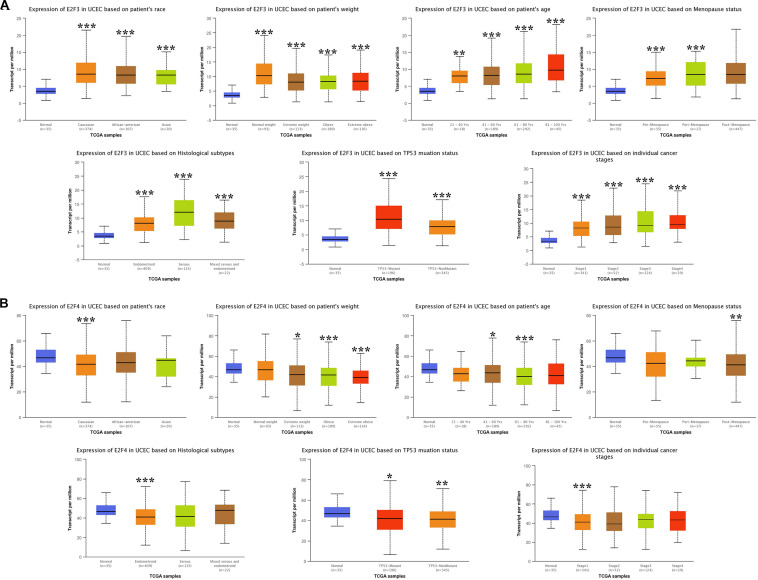
The expression level of E2F3 and E2F4 in UCEC. **(A)** E2F3 expression in multiple subgroups of patients with UCEC. Stratified along race, weight, age, menopause status, histological subtypes, TP53 mutation status, and cancer stage. **(B)** E2F4 expression in subgroups of patients with UCEC, stratified along race, weight, age, menopause status, histological subtypes, TP53 mutation status, and cancer stage. *P < 0.05; **P < 0.01; ***P < 0.001.

**FIGURE 6:**
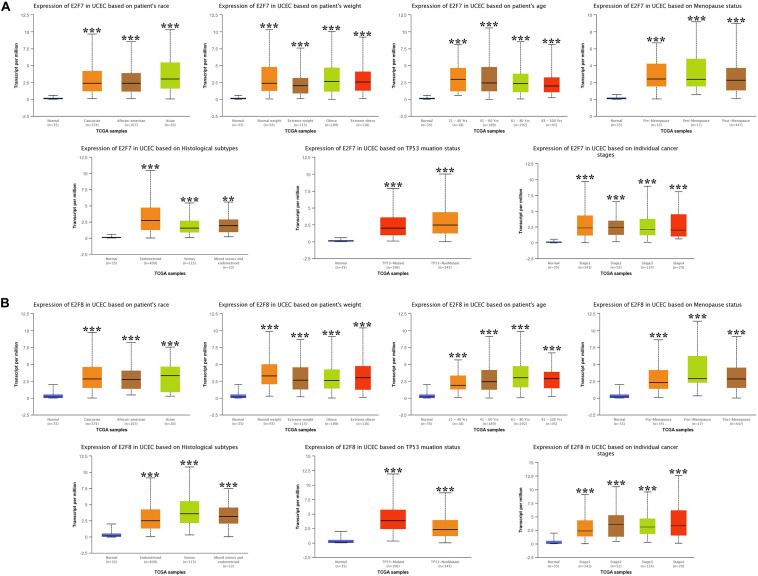
The expression level of E2F7 and E2F8 in UCEC. **(A)** E2F7 expression in multiple subgroups of patients with UCEC, stratified along race, weight, age, menopause status, histological subtypes, TP53 mutation status, and cancer stage. **(B)** E2F8 expression in subgroups of patients with UCEC, stratified along race, weight, age, menopause status, histological subtypes, TP53 mutation status, and cancer stage. *P < 0.05; **P < 0.01; ***P < 0.001).

We then analyzed the association between abnormally expressed E2Fs (E2F1/2/3/4/7/8) and UCEC clinical pathological along race, weight, age, menopause status, histological subtypes, TP53 mutation status, and cancer stage. Here, the transcription of E2F1 (Figure 4A), E2F2 (Figure 4B), and E2F3 (Figure 5A) mRNA was upregulated in UCEC patients compared with healthy individuals in subgroup analyses based on race, weight, age, menopause status, histological subtypes, TP53 mutation status, and cancer stage. Comparable findings were obtained for E2F7 (Figure 6A) and E2F8 (Figure 6B). Moreover, the transcription of E2F4 (Figure 5B) mRNA was downregulated in UCEC patients compared with healthy individuals in subgroup analyses based on race, weight, age, menopause status, histological subtypes, TP53 mutation status, and cancer stage.

### Prognostic Value of E2F Family in UCEC

**FIGURE 7:**
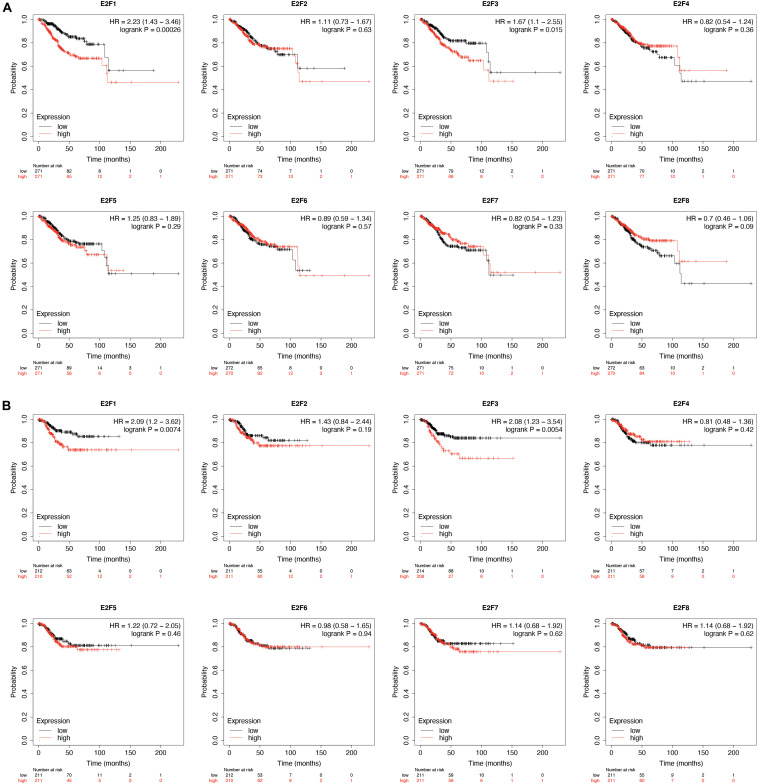
The prognostic value of E2Fs in UCEC. **(A)** The OS curve for E2Fs in UCEC. **(B)** The RFS curve for E2F, in UCEC.

KM-plotter analysis for prognostic value of E2F family in UCEC revealed that UCEC patients overexpressing E2F1 (HR = 2.23, 95%:1.43–3.46, *P* = 0.00026) and E2F3 (HR = 1.67, 95%:1.1–2.55, *P* = 0.015) had a worse OS (Figure 7A). However, the expression level of E2F2, E2F4, E2F5, E2F6, E2F7, and E2F8 had no effect on the OS of these patients (Figure 7A). With regard to RFS, we found UCEC patients expressing E2F1 (HR = 2.09, 95%:1.2–3.62, *P* = 0.0074) and E2F3 (HR = 2.08, 95%:1.23–3.54, *P* = 0.0054) to have a worse RFS (Figure 7B). However, the expression of E2F2, E2F4, E2F5, E2F6, E2F7, and E2F8 had no effect on the RFS of UCEC patients (Figure 7B). Therefore, E2F1 and E2F3 are potential prognostic biomarkers for UCEC.

### The Role of the E2F Family in UCEC

**FIGURE 8:**
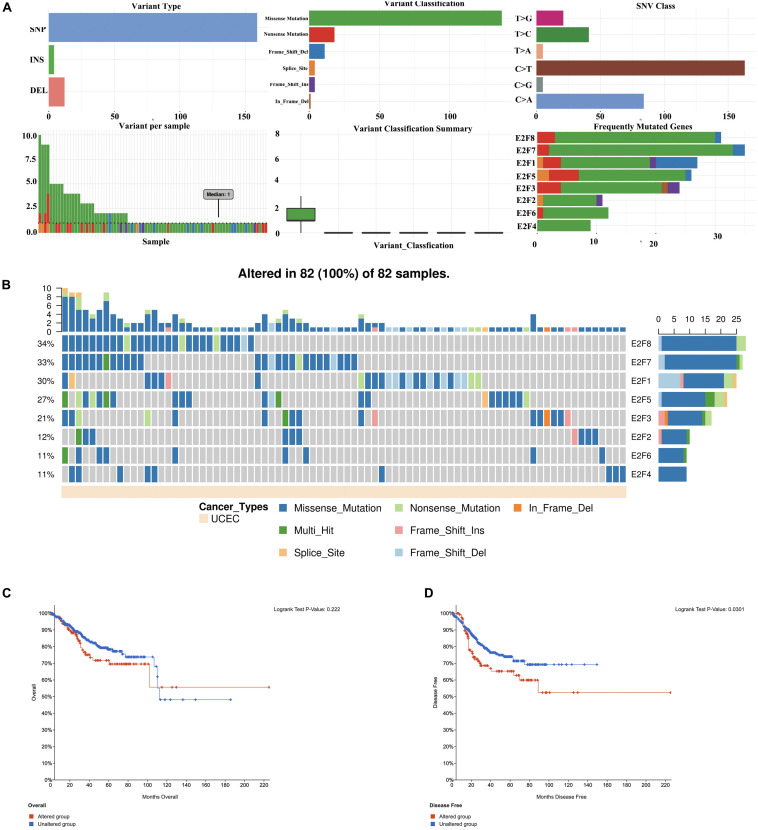
The genetic alteration in E2Fs in UCEC. **(A)** Plot displays for genetic alteration frequency and variant types of E2Fs in UCEC. **(B)** Waterfall plot for the distribution and classification of genetic alteration in E2Fs in UCEC. **(C,D)** The effect of E2Fs on the genetic alteration and corresponding prognosis of UCEC patients.

**FIGURE 9:**
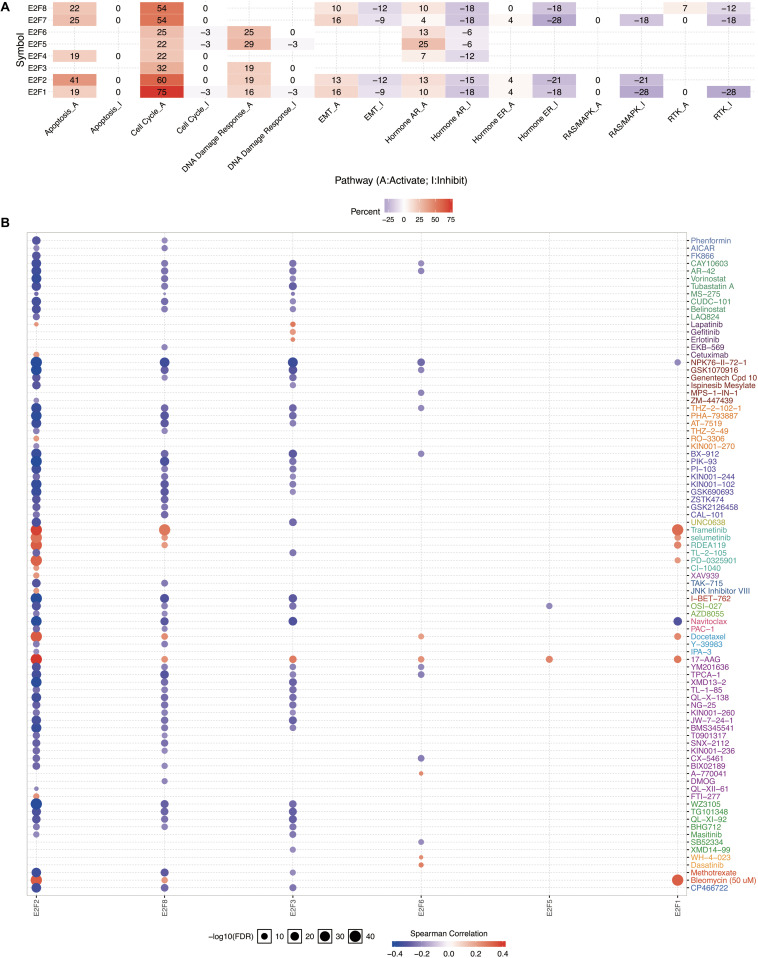
The cancer related pathways and drug resistance analysis of E2Fs in UCEC. **(A)** The association between E2Fs and cancer-related activity. **(B)** The role of E2Fs in drug-sensitivity analysis.

Members of E2F were dysregulated to explore the role of these proteins in UCEC. Follow-up analyses were performed to explore the correlation between the E2F family and the major hallmarks of UCEC. Genetic alteration analysis revealed that the genetic alteration in the E2F genes encompassed missense and non-sense_Mutations. In particular, they included In_Frame_Del, Multi_Hit, Frame_shift_Ins, Spice_Site, and Frame_Shift_Del (Figures 8A,B). Among the E2F family, E2F8, E2F7, and E2F1 were the top three most-frequently altered genes in UCEC (Figures 8A,B). However, these genetic alterations had no effect on the OS (Figure 8C, *P* = 0.222) and disease-free survival (Figure 8D, *P* = 0.0301) of UCEC patients, even though the *p*-value of OS is less than 0.05. Pathway analysis validated the passive role of E2F8, E2F7, and E2F1 in the OS of UCEC patients by revealing that E2Fs were mainly associated with the activation of apoptosis pathways, disruption of cell cycle, and impairment of DNA damage response pathways (Figure 9A). Because the nature of genetic alternations influences clinical treatment intervention, mutation types are potential targets for anticancer drug matching. Meanwhile, drug-sensitivity analysis demonstrated that the expression of E2F2/3/8 negatively correlated with drug resistance (Figure 9B), suggesting the use of E2F2/3/8 as novel markers for drug-sensitivity screening.

### Immune Infiltrations Analysis of the E2F Family in UCEC

**FIGURE 10:**
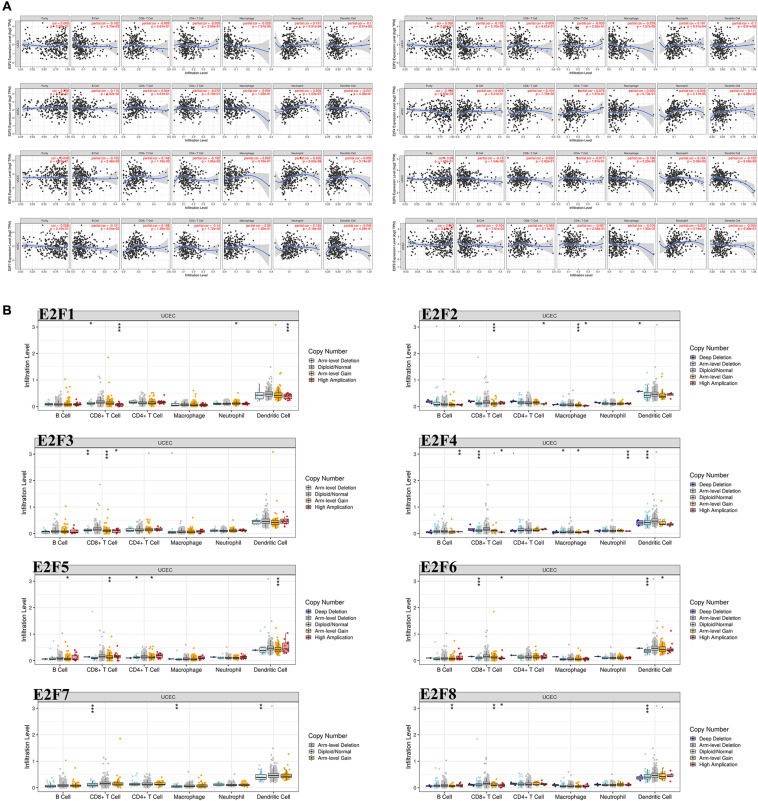
The correlation between E2Fs and immune infiltration in UCEC. **(A)** The correlation between expression of E2Fs and the abundance of immune cells in UCEC. **(B)** The role of somatic copy number alterations in E2Fs and immune cell infiltration in UCEC. *P < 0.05, **P < 0.01, ***P < 0.001.

As shown in Figure 10A, the expression of most members of E2F family was negatively correlated with the abundance of B cell, except for E2F4 and E2F8. That of CD8 + cell positively correlated with the expression of E2F4, E2F5, and E2F7 (Figure 10A). Moreover, the abundance of CD4 + cell negatively correlated with the expression of E2F5 and E2F7 (Figure 10A). As for the macrophage, an abundance of these cell types negatively correlated with the expression of E2F1, E2F2, and E2F6 (Figure 10A). Interestingly, the abundance of neutrophil negative correlated with all members of E2F family (Figure 10A). Moreover, we also found that somatic copy number alterations in the E2F family inhibit immune cell infiltrations (Figure 10B).

### Enrichment Analysis of the E2F Family

**FIGURE 11:**
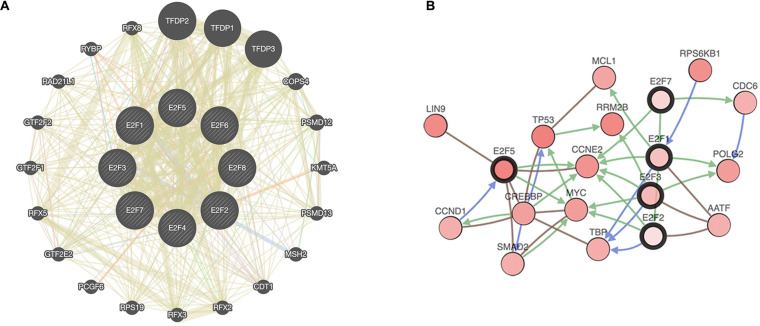
A network of E2Fs with the associated neighboring genes. **(A)** Protein-protein interaction network of E2Fs. **(B)** Gene-gene interaction network of E2Fs.

**FIGURE 12:**
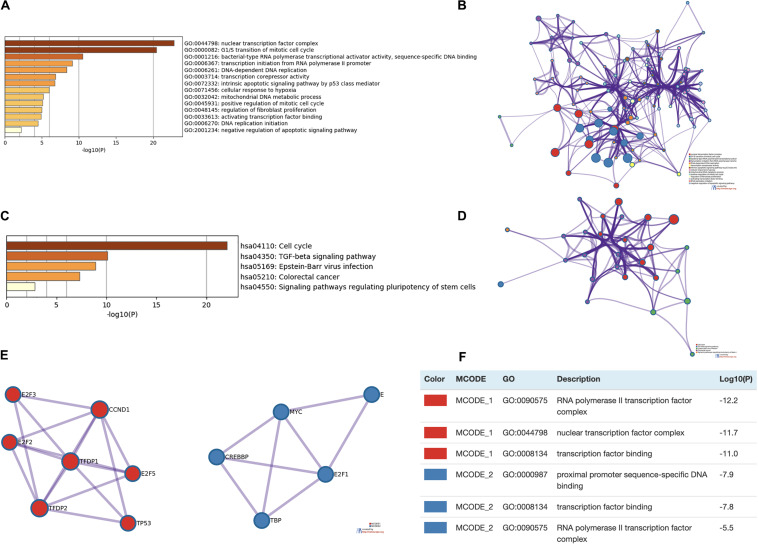
Enrichment analysis of E2Fs and associated genes in UCEC. **(A,B)** Bar graph and network of top 20 enriched items in GO analysis. **(C,D)** Bar graph and network of the significantly enriched items based on KEGG pathways analysis. **(E,F)** Protein-protein interaction network and MCODE components in E2Fs and neighboring genes.

**FIGURE 13:**
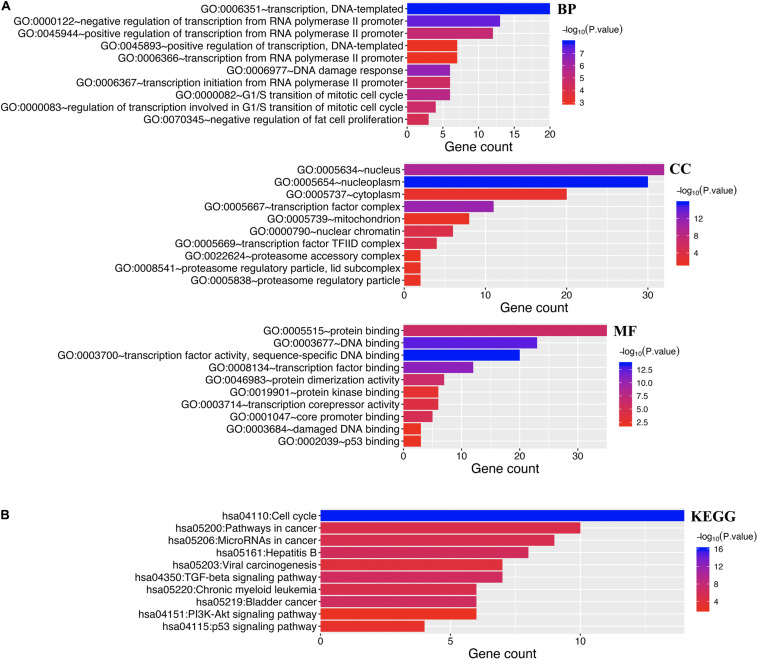
Enrichment analysis of both E2Fs and neighboring genes in UCEC. **(A)** Bar graph of top 10 enriched GO pathways. **(B)** KEGG derived Bar graph of the significantly enriched pathways.

Enrichment analysis was performed to explore the role of E2F in UCEC. We first constructed a network to explore the association between neighboring genes and E2F family. Our findings are shown Figure 11, featuring 34 adjacent genes. GO enrichment analysis in Metascape of E2F family were mainly associated with nuclear TF complex, G1/S transition of mitotic cell cycle, sequence-specific DNA binding, initiation of DNA transcription from polymerase II promoter, DNA-dependent DNA replication, and intrinsic apoptosis signaling pathway mediated by P53 (Figures 12A,B). KEGG pathways analysis demonstrated the involvement of the E2F family in the cell cycle, TGF-beta signaling pathway, Epstein-Barr virus infection, and colorectal cancer (Figures 12C,D). MCODE further revealed that the E2F family and neighboring genes influenced for the binding of RNA polymerase II TF complex, nuclear TF complex, TF binding, and sequence-specific DNA binding (Figures 12E,F). We additionally validated our results using DAVID 6.8. GO enrichment analysis revealed that E2F genes were mainly involved in transcription, DNA-templated, regulation of transcription, DNA damage response, G1/S transition of mitotic cell cycle, protein binding, DNA binding, TF binding, damaged DNA binding, and p53 binding (Figure 13A). As for the KEGG pathways enrichment analysis, our results suggest that E2F participates in cell cycle progression, cancer pathways, viral carcinogenesis, TGF-beta signaling pathway, PI3K-Akt signaling pathway, and p53 signaling pathway (Figure 13B).

### Kinase and miRNA Are Targets for E2F Family in UCEC

We further explored the influence of E2F on kinase and miRNA in UCEC. It was revealed that multiple kinases are targets for E2F (Table 1). In particular, AUPKB and CDK1 are targets for E2F1, whereas CDK1 and CDK2 are targets for E2F2. E2F3 and E2F4 target PLK1 and ATM; CDK2 and LCK, respectively. ATM kinase is targeted by E2F5, E2F6, and E2F7. PLK1 was additionally targeted by E2F5, E2F7, and E2F8. Meanwhile, miRNA targets for E2F family are shown in Table 2. Briefly, miRNA targets for E2F1 include (TATTATA) MIR-374 and (CATTTCA) MIR-203, whereas MIR-199A (CTACTGT) and MIR-137 (AAGCAAT) are targets for E2F2. Moreover, MIR-302C (ATGTTAA) and MIR-381 (CTTGTAT) are miRNA targets for E2F3. As E2F4, it targets MIR-384 (CTAGGAA) and MIR-381 (CTTGTAT), whereas E2F5 targets MIR-154, MIR-487 (GTATGAT), and MIR-302C (ATGTTAA). E2F6 on its part targets MIR-212, MIR-132 (GACTGTT), and MIR-499 (AGTCTTA). Finally, E2F7 targets MIR-522 (ACCATTT) and MIR-508 (TACAATC), with E2F8 targeting MIR-323 (TAATGTG) and MIR-522 (ACCATTT).

**TABLE 1:** The Kinase targets for E2Fs in UCEC.

**E2Fs**	**Kinase Targets**	**LeadingEdgeNum**	**FDR**
E2F1 E2F2 E2F3 E2F4 E2F5 E2F6 E2F7 E2F8	Kinase_AUPKB Kinase_ CDK1 Kinase_CDK1 Kinase_CDK2 Kinase_PLK1 Kinase_ATM Kinase_CDK2 Kinase_LCK Kinase_ATM Kinase_PLK1 Kinase_ATM Kinase_ CDK1 Kinase_ PLK1 Kinase_ ATM Kinase_ CDK1 Kinase_ PLK1	30 78 73 87 32 50 90 16 66 34 41 87 47 50 130 38	*p* < 0.0001 *p* < 0.0001 *p* < 0.0001 *p* < 0.0001 *p* < 0.0001 *p* < 0.0001 *p* < 0.0001 0.003 *p* < 0.0001 *p* < 0.0001 *p* < 0.0001 *p* < 0.0001 *p* < 0.0001 0.002 *p* < 0.0001 *p* < 0.0001

**TABLE 2:** The miRNA targets for E2F in UCEC.

**E2Fs**	**miRNA Targets**	**LeadingEdgeNum**	***P*-value**
E2F1 E2F2 E2F3 E2F4 E2F5 E2F6 E2F7 E2F8	TATTATA, MIR-374 CATTTCA, MIR-203 CTACTGT, MIR-199A AAGCAAT, MIR-137 ATGTTAA, MIR-302C CTTGTAT, MIR-381 CTAGGAA, MIR-384 CTTGTAT, MIR-381 GTATGAT, MIR-154, MIR-487 ATGTTAA, MIR-302C GACTGTT, MIR-212, MIR-132 AGTCTTA, MIR-499 ACCATTT, MIR-522 TACAATC, MIR-508 TAATGTG, MIR-323 ACCATTT, MIR-522	104 100 53 68 81 92 70 17 34 35 137 21 71 26 70 79	*p* < 0.0001 *p* < 0.0001 *p* < 0.0001 *p* < 0.0001 *p* < 0.0001 *p* < 0.0001 *p* < 0.0001 *p* < 0.0001 *p* < 0.0001 *p* < 0.0001 *p* < 0.0001 *p* < 0.0001 *p* < 0.0001 *p* < 0.0001 *p* < 0.0001 *p* < 0.0001

## Discussion

E2Fs in a heterogeneous family of TFs that can regulate DNA damage response and transition between cell cycles, aiding in cancer development and metastasis (Kent and Leone, 2019). Increasing evidences have demonstrated the significance of E2Fs in the prognosis of certain malignancies. However, its role in the tumorigenesis, metastasis, and prognosis of EC has not been explored. This study therefore discerned this relationship.

We first detected the expression pattern of E2Fs in EC. E2F1/2/3/7/8 were found to be upregulated in EC tissues, converse to downregulated E2F4. Further prognostic analysis validated the potential role of E2F1 and E2F3 as prognostic biomarkers for EC. The prognostic property of E2Fs is, however, not a new phenomenon. Indeed, E2F2/5/8 have been shown to be novel biomarkers for prognosis for ovarian cancer (Zhou et al., 2019), E2F1/2/5/8 for lung cancer (Sun et al., 2018), and multiple members of E2F for gastric and breast cancer (Manicum et al., 2018; Sun et al., 2019).

In addition to the above, the alterations in E2F genes are associated with several common cancer-related pathways. In brief, this study found that E2Fs activate apoptosis pathways, modulate cell cycle, and impair DNA damage response pathways. These finding are comparable to previous studies, where E2Fs were reported to be highly regulated throughout the cell cycle and apoptosis (Benevolenskaya and Frolov, 2015). A separate study also reported that most E2Fs are upregulated by both transcriptional and post-transcriptional methods in response to DNA damage (Kent and Leone, 2019). Moreover, we uncovered an association between E2Fs and viral carcinogenesis and TGF-beta, PI3K-Akt, and p53 signaling pathways, all involved in the proliferation and invasion of tumor cells. Therefore, inhibition of E2Fs may regulate the tumorigenesis and progression of EC *via* these pathways.

Our study also revealed the association between E2Fs and immune cell infiltrations, including CD8 + cell, CD4 + cell, macrophages, and neutrophils. Indeed, there are limited studies on the association between E2F family and immune infiltrations. However, Shom et al. found that E2Fs could regulate the effects of CDK4/6 inhibitors on regulatory T cells (Goel et al., 2017), suggesting the regulatory role of E2F1 on immune responses with regard to antitumor immunity (Ren et al., 2014). Another study also reported a (negative) correlation between E2F3 expression and immune cell infiltration (Deng et al., 2020).

This study revealed further several kinase targets for several members of E2F family in EC. Briefly, CDK1, CDK2, PLK1, and ATM are targets for most of E2F family. These kinases play a vital part in genomic stability and regulation of cell cycle (Herrera et al., 2018). Surprisingly, ATM kinase could induce DNA damage in EC (Takeuchi et al., 2019). ATM is thought to regulate the chromosomal instability as well as promote anti-tumor effect by inducing DNA damage and killing of tumor cells (Drosos et al., 2017; Maciejowski and de Lange, 2017). Therefore, E2Fs family may modulate DNA repair and impair cell cycle progression in EC *via* ATM kinase.

Our result also identified several miRNAs such as MIR-522, MIR-302C, and MIR-381 associated with expression of E2Fs. Several of these miRNA targets were found to be diagnostic and prognostic markers for EC, and participate in tumor invasion, apoptosis, and drug resistance. Our previous study demonstrated that MIR-302C could suppress EMT and induce apoptosis in EC (Li et al., 2018). In the same cancer type, MIR-381 could suppress proliferation and invasion of tumor cells (Tu et al., 2018). Therefore, regulation of these miRNAs by E2F exacerbate tumorigenesis and progression of ECs.

Our study has some limitations. Analysis on the transcriptional level can reflect some aspects of the function of E2Fs in EC, but not global changes. Moreover, another independent cohort and more *in vitro* or *in vivo* studies should be performed to validate our results.

## Conclusion

Taken together, our study explicitly explored the expression of E2Fs and their clinical significance with regard to EC. Findings of this research expand our knowledge on the role of E2Fs, with reference to EC.

## Data Availability Statement

All datasets presented in this study are included in the article/ Supplementary Material.

## Ethics Statement

The studies involving human participants were reviewed and approved by the Scientific Research and New Technology Ethical Committee of the Shengjing Hospital of China Medical University. Ethical number: 2018PS251K. The patients/participants provided their written informed consent to participate in this study.

## Author Contributions

YZ performed conceptualization, methodology, validation, and writing - original draft. ZW performed conceptualization. JM performed resources. JH and YL performed investigation. YW and HC performed visualization. LS performed writing - review and editing. XM performed conceptualization, funding acquisition, and writing - review & editing. All authors contributed to the article and approved the submitted version.

## Conflict of Interest

The authors declare that the research was conducted in the absence of any commercial or financial relationships that could be construed as a potential conflict of interest.

## Supplementary Material

Supplementary Figure 1 The flow chart of analyses.

Supplementary Table 1 Primer sequences for qRT-PCR.
