# Supplementary figures and images for: Bioinformatics Identification of the Expression and Clinical Significance of E2F Family in Endometrial Cancer
Source: Front Genet. 2020 Nov 4;11:557188. doi: 10.3389/fgene.2020.557188 (PMC7672218; doi:10.3389/fgene.2020.557188)

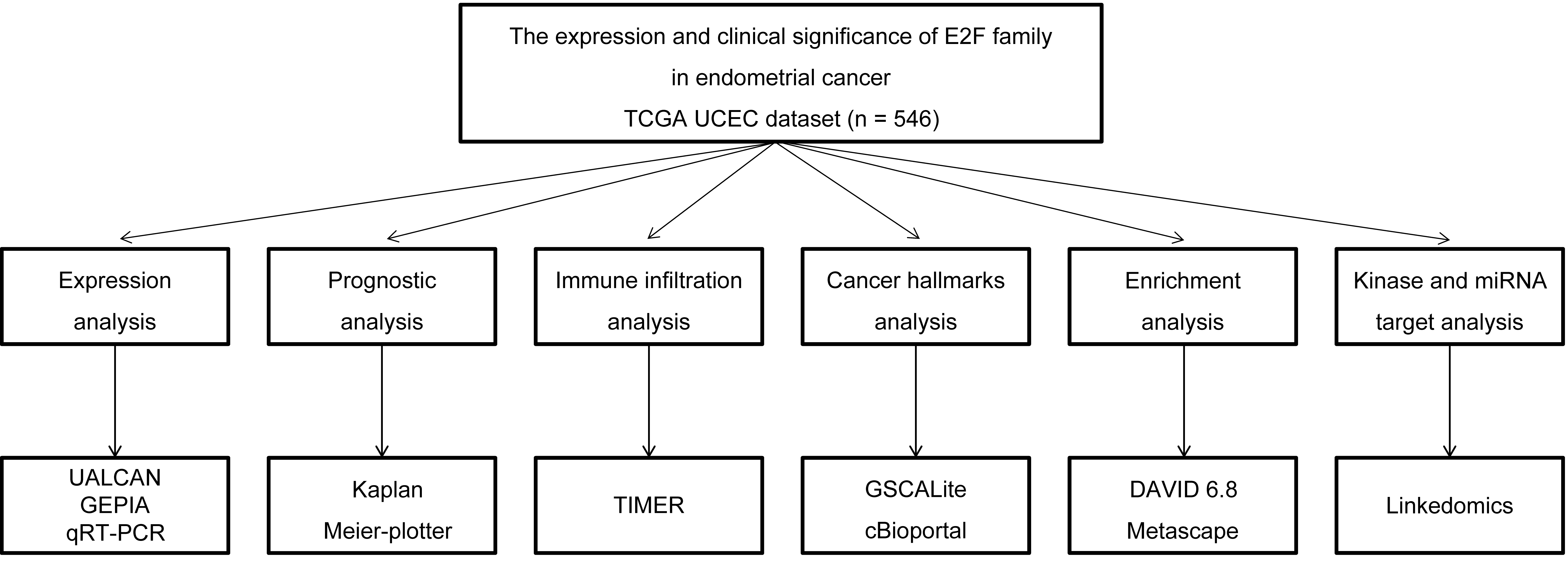

Supplement: Supplementary Figure 1 — The flow chart of analyses. [file Image_1.TIF]
