# Supplementary material for: Bioinformatics Identification of the Expression and Clinical Significance of E2F Family in Endometrial Cancer
Source: Front Genet. 2020 Nov 4;11:557188. doi: 10.3389/fgene.2020.557188 (PMC7672218; doi:10.3389/fgene.2020.557188)
Supplement: Supplementary Table 1 — Primer sequences for qRT-PCR. [file Table_1.docx]

| Primer | Forward | Reverse |
| --- | --- | --- |
| E2F1 | AGCGGCGCATCTATGACATC | GTCAACCCCTCAAGCCGTC |
| E2F2 | CTGAAGGAGCTGATGAACACG | CCCTTGGGTGCTCTTGAGATA |
| E2F3 | GTCATCAGTACCTCTCAGATGG | GCAGACCAAGAGACGTATCATA |
| E2F4 | GAAATCTTTGATCCCACACGAG | ACTCTCGTCCAGGTTGTAGATA |
| E2F5 | CTCACTACCAAGTTCGTGTCG | TTTTGCCTCACAGCCAAAGTAT |
| E2F6 | AGGAGCACCAACGGACCTATCG | GTCCCGACACCTTCAGACCTTTTG |
| E2F7 | ACCCTCAGATTCCACAGACC | AGTTTGCTGTTGCCTTTCCT |
| E2F8 | GAAATCCCAACCAAGTCGAA | CTTCGTCAAGGCAGATGTCA |
| GAPDH | GCACCGTCAAGGCTGAGAAC | TGGTGAAGACGCCAGTGGA |

Supplementary Table 1: Primer sequences for qRT-PCR.
